# Editorial: Molecular selection of promising resistant genotypes to disease in cereal crops

**DOI:** 10.3389/fpls.2026.1808886

**Published:** 2026-03-31

**Authors:** Amira M. Mourad, Andreas Börner

**Affiliations:** 1 Leibniz Institute of Plant Genetics and Crop, Plant Research (IPK), Corrensstrasse 3, D−06466 Seeland, OT Gatersleben, Gatersleben, Germany; 2 Agronomy Department, Faculty of Agriculture, Assuit University, Assuit, Egypt

**Keywords:** disease resistance, genome-wide association studies (GWAS), marker-assisted selection, molecular breeding, Quantitative Trait Loci (QTL)

Cereal crops are essential to the global food system because they provide a food supply. However, there is always the threat of crop failure due to disease. With modern fungal and bacterial pathogens, crop failure could be considered as an expected risk due to climate change, new pathogen strains, and changes in farming practices. Developing cultivars that carry durable disease resistance is becoming more critical (Bhavani et al., 2021; Mourad et al., 2024). With traditional breeding approaches, there are always reservations due to environmental selection and the varied resistance traits that are masked by other phenotypes. However, new advances in molecular breeding and the use of genomic tools are allowing breeders to create more robust molecular selection tools to assess the breeding values of plants and speed up the selection of desirable plants (Rasheed and Xia, 2019). Improvements in molecular genetics have changed the methods of identifying and deploying disease resistance in cereal crops from phenotype alone to a more integrated approach regarding the genetic and molecular information and the relevant functional diversity. Recent studies show resistance deriving from both major effect loci and multifaceted regulatory systems governed by the interactions among the host, the pathogen, and the genetic background (Arora et al., 2019). The incorporation of molecular selection in breeding cereals has become important for building consistent disease resistance that lasts over time (Eltaher et al., 2021).

This Research Topic brings together four original articles that collectively highlight the power of integrating phenotypic evaluation with molecular, genomic, and transcriptomic approaches to identify and characterize disease resistance in major cereal crops. The contributions span multiple species, diseases, and methodological frameworks, providing a comprehensive perspective on modern strategies for resistance breeding.

Kushwaha et al. and Zhang et al. emphasized genetic mapping and functional genomics on disease resistance in rice, respectively. The *qBK1.2* is a major quantitative trait locus for resistance to bakanae disease, a devastating seed-borne disease of rice, that is difficult to select through traditional breeding. The high-resolution mapping of *qBK1.2*, delemited the locus to a precise genomic interval and laid the groundwork for the identification of candidate genes and marker-assisted selection. This work is a significant contribution to the incorporation of alleles for resistance to bakanae disease in rice breeding and exemplifies the contribution of refined QTL mapping to the analysis of complex traits for disease resistance. Zhang et al. conducted a transcriptome analysis of resistant and susceptible near isogenic lines infected by *Xanthomonas oryzae* pv. *oryzicola* the causal agent of bacterial leaf streak, lead to the characterization of the resistant response. The molecular pathways of the responsive resistant response of the host illustrated the molecular interactions of the host and the pathogen. This functional characterization provide a valuable resource for the identification of candidate genes underlying disease resistance, elucidation of their molecular mechanisms, and the development of tightly linked molecular markers to accelerate marker-assisted selection in breeding programs.

As a staple food crop, wheat has great global significance. Mourad et al. illustrates this significance, focusing on the resistance to powdery mildew in Egyptian wheat. Using molecular techniques, the authors discovered new seedling resistance genes on chromosome 5B. These new resistance loci are crucial due to the fast development of powdery mildew pathogens and the high frequency of previously deployed resistance genes getting overcome. Moreover, the selection of resistant genotypes based on the phenotypic response, number of significant alleles associated with the resistance, and the genetic distance between the selected genotypes demonstrated the efficiency of combining marker-assisted selection and phenotypic analysis. This research contributes to the growing number of resistance sources wheat breeders can draw on and emphasizes the need to determine sources of resistance *in situ* to local environment and pathogen pressures.

Along with rice and wheat, this Research Topic examines disease resistance in other cereal crops, focusing on the genetic and phenotypic complexity. A genome-wide study of a novel sweet sorghum diversity panel assessed genetic diversity, population structure, and resistance levels to anthracnose disease. Through phenotypic and genetic analysis, Cuevas et al. discovered loci associated with resistance and illustrated the applicability of various germplasm panels in identifying beneficial alleles. These results advocate for the positive impact of employing genetic diversity in breeding programs focused on improving disease resistance and other favorable agronomic traits.

The four articles in this Research Topic collectively demonstrate that breeding disease-resistant cereals benefits from integrating approaches such as QTL mapping, genome-wide association studies, and transcriptome analysis. The identification and characterization of resistance loci, candidate genes, and defense-related pathways provide valuable resources for marker-assisted and genomic selection. These studies advance our understanding of the genetic and molecular bases of disease resistance in cereals and help frame the challenge of developing resilient cultivars. They highlight that combining molecular tools with thorough phenotypic evaluation accelerates the identification and integration of promising resistant genotypes. Such integrated strategies will be essential for sustaining cereal production and ensuring global food security amid evolving disease pressures.
